# Conching of dark chocolate – Processing impacts on aroma-active volatiles and viscosity of plastic masses

**DOI:** 10.1016/j.crfs.2024.100909

**Published:** 2024-10-31

**Authors:** Yvonne Guckenbiehl, Aurora Magdalena Morales Romero, Helen Haug, Eva Ortner, Isabell Rothkopf, Ute Schweiggert-Weisz, Andrea Buettner, Susanne Gola

**Affiliations:** a Fraunhofer Institute for Process Engineering and Packaging IVV, Giggenhauser Str. 35, 85354, Freising, Germany; b Chair of Aroma and Smell Research, Department of Chemistry and Pharmacy, Friedrich-Alexander-Universität Erlangen-Nürnberg, Henkestr. 9, 91054, Erlangen, Germany; c Technical University of Munich, School of Life Sciences, Plant Proteins and Nutrition, Weihenstephaner Berg 1, 85354, Freising, Germany

**Keywords:** Plastic conching, Conching parameters, Chocolate flavor, Aroma distribution, Gas chromatography-mass spectrometry, Closed cavity rheometer

## Abstract

The conching process plays a key role in determining the sensory and rheological properties of dark chocolate. To further understand this process, changes in the chocolate mass during plastic conching were investigated on a time-resolved basis with varying conching temperature, shear direction, and with or without the presence of residue from previous trials (pre-charge) on the conche vessel wall. Six selected odorants (acetic acid, benzaldehyde, linalool, 2,3,5,6-tetramethylpyrazine, 2-phenylethanol, 2-phenylethyl acetate) were quantified in fat and particle phases of chocolate masses. Particularly at elevated conching temperature, the odorant concentrations were found to decrease (up to 78.0% in the fat phase). The highest concentrations of desired odorants were determined mostly after conching without pre-charge. During conching, odorants were observed to accumulate increasingly in the fat phase (up to 91.7%) with decreasing odorant polarity. Similarly, it was found that conching temperature and the absence of pre-charge had the highest impact on the rheological properties of the chocolate mass, resulting in lowest and highest complex viscosity, respectively. In conclusion, some positive outcomes of conching, namely the retention of desired odorants and the reduction of viscosity, were inversely related at elevated temperature or in the absence of pre-charge, necessitating compromises to achieve optimal flow properties and flavor. Our results contribute to a deeper understanding of the influence of conching on the quality of dark chocolate by providing insights into the complexity of aroma migration and rheological changes during conching.

## Highlights

Less polar odorants accumulated increasingly in the fat phase during conching
Increased conching temperature reduced compound concentrations and viscosity the most
The complex viscosity was highest after conching without pre-charge
Highest desired odorants concentration in fat phase was obtained without pre-charge

## Graphical abstract

**Figure:**
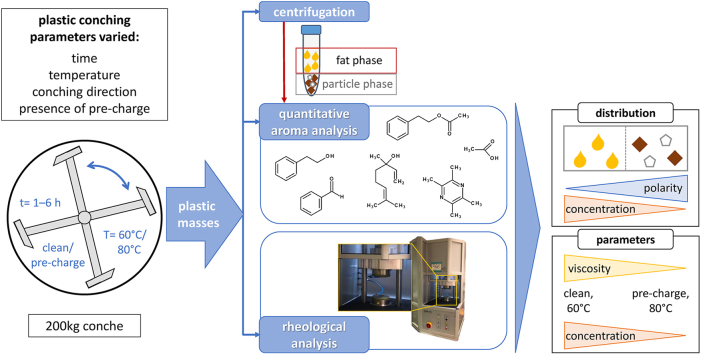

## 1 Introduction

Dark chocolate is a suspension of cocoa and sugar particles dispersed in a continuous fat phase (cocoa butter), offering unique sensory properties that make it a popular food all over the world. Its characteristic aroma, taste, and texture are achieved, *inter alia*, by conching, which is an essential step in the chocolate production process after cocoa bean fermentation, drying, and roasting. The conching process starts with a refined mixture of sugar and cocoa liquor and can be described as a mixing, shearing, tempering, and aerating of the dry and inhomogeneous starting material until a homogeneous flowing melt with harmonious aroma properties is obtained (Ziegleder, 2017). Furthermore, excess moisture is removed during conching, which is crucial for achieving suitable flow properties of the final product for further processing and may support the aroma development throughout the process (Beckett, 2003; Afoakwa et al., 2008b). The conching process can be divided into three phases that describe the texture changes during the process: the dry conching, the plastic conching, and the liquid conching (Biehl and Ziegleder, 2003). The dry conching describes the beginning of the process as the refined material turns into a firmer, plastic mass and the plastic conching phase starts. The plastic conching is the most relevant phase for texture and flavor changes (Albak and Tekin, 2016; Guckenbiehl et al., 2022) since the strongest shear forces are attained within this conching phase (Danzl and Ziegleder, 2014). After adding cocoa butter and/or emulsifier, the liquid conching phase starts and the plastic mass turns into a homogeneous, liquid melt.

The texture changes during conching are mainly attributed to de-agglomeration effects (Hoskin and Dimick, 1980). After refining the mixed ingredients, the obtained flakes consist of agglomerates that are built from solid sugar and cocoa particles entrapping the fat and leaving the starting material for conching as a dry powder. During conching, these agglomerates are broken up due to the thermomechanical treatment inside the conche and the enclosed fat is released. Solid particles are thus coated by fat over time and the solid-solid interactions are reduced as the particles are embedded in the continuous fat phase (Bolenz et al., 2003; Vivar-Vera et al., 2008; Ziegleder, 2017). Consequently, the viscosity of the mass decreases with increasing conching duration (Vivar-Vera et al., 2008; Guckenbiehl et al., 2022). These texture changes during conching depend on the ingredients used, the process parameters, and the water content of the raw materials. Urbańska et al. (2021), for example, determined a significant influence of the conching time (1 h versus 3 h) on the Casson viscosity in milk chocolates when conched at 50 °C and at 55 °C. However, the effect of conching time was not significant when conched at 60 °C, which was explained by the fact that an increased conching temperature resulted in a faster attainment of a lower viscosity. Further, Afoakwa et al. (2008a) investigated the influence of chocolate composition and particle size distribution on the rheological properties of finalized dark chocolate. The authors found a decrease in viscosity with increasing particle size or raising fat and lecithin contents. However, the influence of various conching parameters on the rheological characteristics of dark plastic mass has not yet been studied in detail. Understanding texture changes in conjunction with the aroma development during conching holds the potential to optimize the process in a targeted manner.

After conching, the chocolate mass is described by a mellow and balanced flavor perception (Fischer et al., 2010; Toker et al., 2020; Ziegleder, 2017). The change of flavor is possibly achieved by various mechanisms occurring during the conching process. Apart from the texture change, the change in aroma concentration and distribution is crucial for the perceived aroma of the final chocolate (Fischer et al., 2010). Aroma-relevant changes during conching are supposed to underlie evaporation, re-distribution, and formation (Counet et al., 2002; Ziegleder et al., 2003; Owusu et al., 2012; Albak and Tekin, 2016). Particularly undesired aroma compounds, such as excess acetic acid and other short-chain acids that could negatively affect the chocolate flavor, are removed by evaporation (Owusu et al., 2012; Engeseth and Ac Pangan, 2018; Augusto and Bolini, 2022). However, desired compounds are removed during conching as well (Counet et al., 2002; Owusu et al., 2013; Ziegleder, 2017). Apart from aroma evaporation, specific compounds were found in increased amounts after conching. Counet et al. (2002) determined 2-phenyl-5-methyl-2-hexenal in significantly higher amounts after conching suggesting a formation by chemical reactions from available aroma compounds. Several pyrazines, such as 2,3,5,6-tetramethylpyrazine (TMP), were found by the authors in significantly elevated levels as well. A third mechanism of aroma development was proposed by Ziegleder et al. (2003), who suggested that the aroma refinement achieved by conching is based on a re-distribution of aroma compounds between cocoa constituents (cocoa particles and cocoa butter) and sugar. Fischer et al. (2010) stated that the aroma perception is mainly driven by the aroma-active volatiles contained in the fat phase. In the fat phase, the authors determined decreasing concentrations of selected aroma compounds with increasing conching time, while the concentrations in the sugar and protein part as well as the cocoa solids were found to remain constant. Although the literature provides some information on the influence of conching on the development of aroma-active compound concentrations in dark chocolate, investigations on the influence of varying recipes and processing parameters on the aroma development are rare. Afoakwa et al. (2009), for example, showed a reduced volatility of lipophilic aroma compounds when the chocolate matrix exhibited a higher fat content, and Owusu et al. (2012) as well as Fischer et al. (2010) tracked the concentrations of selected odorants depending on conching time. Apart from conching duration, the influence of other process parameters, e.g. conching direction and temperature, on aroma-active volatiles are insufficiently addressed in the literature so far.

The objective of this study was to investigate the influence of conching time, temperature, conching direction (with asymmetrically shaped shear elements), and the presence of residual pre-charge on the texture changes, the concentration, and the distribution of aroma-active volatiles within a dark chocolate matrix during plastic conching. Texture changes were captured by measuring the rheological properties of differently conched plastic masses using a closed cavity rheometer (CCR). The aroma development was investigated by determining the concentrations of six selected aroma-active volatiles (acetic acid, benzaldehyde, linalool, TMP, 2-phenylethanol, 2-phenylethyl acetate) in the fat phase and total plastic mass of differently conched masses via stable isotope dilution analysis (SIDA) and gas chromatography-mass spectrometry (GC-MS).

## 2 Materials and methods

### 2.1 Production of sample masses

Plastic conched masses were produced based on a standard recipe for dark chocolate (60 g/100 g cocoa content) containing 45 g/100 g cocoa liquor, 40 g/100 g sugar, and 15 g/100 g deodorized cocoa butter. Cocoa liquor and sugar were mixed and refined by an industrial partner to obtain flakes (29 g/100 g fat content) from one batch. The flakes were used for conching experiments on a time-resolved basis in a single shaft conche with a capacity of 200 kg (ELK Bühler, Uzwil, Switzerland). Approximately two weeks elapsed from the production of the flakes to the conching experiments. In the meantime, the flakes were stored chilled in airtight white plastic buckets, each holding 20 kg. During all experiments, the dry conching phase was kept consistent for 30 min at 50 °C and rotation direction forwards at 500 rpm. Dry and plastic conching were generally performed keeping the lid open to ensure ventilation of the conche vessel. The plastic conching phase was performed at 1200 rpm with varying conching parameters including different temperatures and rotation direction of the shear elements (conching direction), and presence of pre-charge (Table 1). Samples of plastic masses were taken in dependence of the conching time by pausing the conche every hour and drawing samples from the plastic mass after 1 h, 2 h, 3 h, 4 h, 5 h, and 6 h of conching. Liquid conching was performed after adding deodorized cocoa butter with closed lid at 50 °C and a rotation speed of 2400 rpm for 15 min forwards, followed by 15 min backwards. According to industrial standards, the conche was then emptied and the residues were left as pre-charge inside the conche. Consequently, the first experiment (noPC_60_for) was performed in an empty conche without pre-charge, while the other experiments were executed with pre-charge from the previous trials.

**Table 1:** Conching parameters applied during conching experiments on a 200 kg scale.

designation	temperature [°C]	direction of rotation	pre-charge	time [h]
noPC_60_for	60	forwards	no	1–6
PC_60_for	60	forwards	yes	1–5.5^a^
PC_80_for	80	forwards	yes	1–6
PC_60_back	60	backwards	yes	1–6

a Performed for a shorter conching time due to time limits but given as 6 h in the following text to increase readability.

### 2.2 Rheological characterization of plastic masses

Rheological characterization was achieved by using a CCR (RPA flex, TA Instruments, New Castle DE, USA) as it was described in detail previously (Guckenbiehl et al., 2022). In brief, measurements were carried out using 5 g (±0.1 g) of the masses plastic conched for 1 h, 3 h, and 6 h (section 2.1). During the measurements, the pressure was set to 450 kPa. Frequency sweep measurements were conducted to determine the complex viscosity (ƞ∗) of the plastic masses as a function of oscillation frequency. The strain was kept constant (γ = 1%) as the oscillation frequency increased gradually (f = 0.1–10 Hz, angular frequency *ω* = 0.628–62.8 rad s^−1^). Throughout the measurements, a temperature of 60 °C was maintained inside the cavity. Measurements were carried out in duplicate.

### 2.3 Aroma analysis of plastic masses

#### 2.3.1 Chemicals and references used for aroma analysis

Dichloromethane (DCM) used for solvent extraction and anhydrous sodium sulfate were purchased from Merck KGaA (Darmstadt, Germany). Prior to use, DCM was purified by distillation and anhydrous sodium sulfate was dried over night at 100 °C. Isotope-labeled standards were used as references for quantification and purchased from Sigma Aldrich (Steinheim, Germany): [^13^C_2_]-acetic acid; aromaLab (Martinsried, Germany): [^2^H_5_]-benzaldehyde, [^2^H_4-5_]-(R,S)-(±)-linalool, [^2^H_12_]-TMP, [^2^H_5_]-2-phenylethyl acetate, and CDN isotopes (Pointe-Claire, France): [^2^H_5_]-2-phenylethanol. Unlabeled references were acquired from Sigma Aldrich (Steinheim, Germany): acetic acid, benzaldehyde, (R,S)-(±)-linalool, TMP, 2-phenylethanol and methyl octanoate, and from Fluka (Seelze, Germany): 2-phenylethyl acetate.

#### 2.3.2 Quantification of selected odorants in fat and particle phase

In order to investigate the influence of conching parameters on aroma concentrations and the distribution of acetic acid, benzaldehyde, linalool, TMP, 2-phenylethanol, and 2-phenylethyl acetate within the chocolate matrix, the compound concentrations were determined in the fat and particle phase of the differently conched plastic masses. Plastic masses were centrifuged at 17 000 ∗*g* and 40 °C for 60 min (6–16K Sigma Laborzentrifugen GmbH, Osterode am Harz, Germany) and decanted afterwards to obtain the fat phase. Subsequently, the fat phase was used for aroma analysis. The concentration in the particle phase (cocoa/sugar particles) could not be measured directly. Therefore, the plastic mass was analyzed aroma-analytically and the proportional concentrations in the particle phase were subsequently calculated by using the following equation:

(1) c_total_ = p_fat_ · c_fat_ + (1 - p_fat_) · c_part_

(2) c_part_ = (c_total_ – p_fat_ · c_fat_) · (1 - p_fat_)^−1^

With c_part_: absolute concentration of selected odorants in particle phase [ppb]

c_total_: concentration of selected odorants in plastic mass [ppb]

c_fat_: absolute concentration of selected odorants in fat phase [ppb]

p_fat_: proportion of fat phase in the plastic mass [%]

Fat phase and plastic mass concentrations of the selected aroma compounds were quantified applying odorant isolation and SIDA as described in the following sections 2.3.3, 2.3.4.

#### 2.3.3 Isolation of selected odorants from plastic masses

Aroma-active volatiles were isolated from the plastic masses by solvent extraction. Samples of plastic masses were extracted four times with DCM. In the first extraction cycle, 1–2 g of the sample was extracted with 75 mL DCM in a closed Erlenmeyer flask (200 mL). Depending on the concentrations of the selected compounds known from prior quantifications, respective isotope-labeled standards were spiked into the solution in known quantity. The mixture was stirred at room temperature for 45 min and subsequently filtered under vacuum (IKA VACSTAR, IKA®-Werke GmbH & Co.KG, Staufen, Germany) by using a Büchner funnel. In the following three extraction cycles, the filtration residue and the filter paper were extracted another 10 min with 75 mL of DCM and subsequently filtered under vacuum. The four extracts were combined, concentrated to an approximate volume of 100 mL by Vigreux distillation at 50 °C, and used for Solvent Assisted Flavour Evaporation (SAFE) to separate volatiles from non-volatiles (Engel et al., 1999). The obtained distillate was dried over anhydrous sodium sulfate, filtered, and further concentrated to a volume of 150 μL by Vigreux and micro distillation at 50 °C.

Samples of fat phases were prepared by a single extraction cycle. 1–2 g of the molten sample and 50 mL of DCM were spiked with isotope-labeled standards and stirred in a closed Erlenmeyer flask (100 mL) for 90 min at room temperature. The extract was directly used for high vacuum distillation applying the SAFE technique. Subsequently, the distillate was dried over anhydrous sodium sulfate, filtered, and concentrated analogously to a final volume of 150 μL.

#### 2.3.4 Quantification of odorants by SIDA and GC-MS

The distillates (2.3.3.) were analyzed applying GC-MS. The analyzing system consisted of a Trace GC Ultra (Thermo Fisher, Waltham MA, USA) coupled to a DSQ II mass spectrometer (Thermo Fisher). A deactivated (DPTMDS) precolumn (5 m × 0.53 mm; Chromatographie Zubehör Trott, Kriftel, Germany) and a DB-FFAP fused silica capillary column (30 m × 0.25 mm, 0.25 μm; Agilent Technologies, Santa Clara CA, USA) were used. The Helium flow was held constantly at 2.2 mL min^−1^ and the sample aliquots (2 μL) were injected automatically by an MPS autosampler (Gerstel, Mühlheim an der Ruhr, Germany) applying the cold on-column technique (40 °C). For separation, the GC oven temperature was initially held at 40 °C for 2 min, was subsequently risen to 235 °C with a heating rate of 8 °C min^−1^, and held for 5 min. Mass spectrometric detection was conducted in electron ionization (EI) mode at 70 eV in full scan mode (*m/z* 35–249). For quantification, binary mixtures of each isotope-labeled standard and the unlabeled analyte were created in five different mass ratios to obtain a response curve. The concentration of each analyte was calculated based on the response curve. Selected ions (*m/z*) of each isotope-labeled standard and analyte are listed in the supplementary information (Table A.1).

### 2.4 Statistical analysis

The determined complex viscosity and odorant concentrations were statistically analyzed via one and four factorial ANOVA followed by a Tukey HSD test.

Samples of the fat phase were analyzed in triplicate and results are given as mean ± standard deviation. Plastic mass samples were analyzed in duplicate, therefore results are given as mean ± range. As the concentration of aroma compounds in the particle phases was calculated (equations (1), (2))), standard deviation from the proportional mean was determined by error propagation based on analytical results determined in the fat phase and plastic mass using equation Eq. A.1 (supplementary information).

Two-sample *t*-test (two-tailed) was conducted to analyze statistically significant differences between the proportions of each odorant in the fat phase at 1 h and 6 h of conching. Analysis was performed via Epitools (Ausvet, Fremantle WA, Australia) based on the mean, standard deviation, and the number of samples (n).

## 3 Results

### 3.1 The complex viscosity of differently conched plastic masses

The impact of different conching parameters on the complex viscosity of plastic masses during dark chocolate conching was investigated using a CCR, which was shown to be a promising method for the rheological characterization of plastic masses in our previous work (Guckenbiehl et al., 2022). Frequency sweep tests were conducted to analyze the masses conched for 1 h, 3 h, and 6 h.

**Fig. 1:**
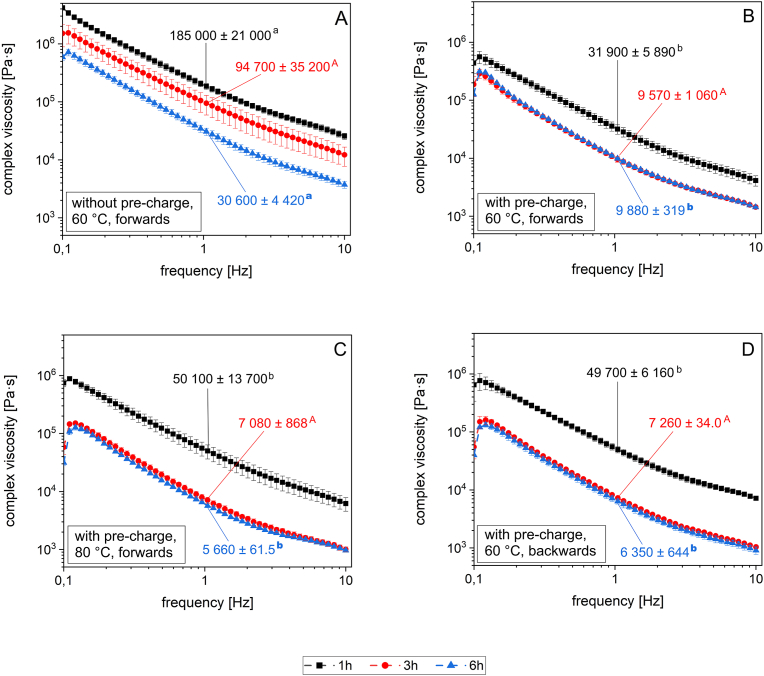
Complex viscosity of plastic mass after conching for 1 h, 3 h, and 6 h applying varying conching parameters. Masses were conched without pre-charge at 60 °C forwards (**A**), with pre-charge at 60 °C forwards (**B**), with pre-charge at 80 °C forwards (**C**), and with pre-charge at 60 °C backwards (**D**). The complex viscosity of plastic masses was determined by frequency sweep tests, is additionally given at f = 1.048 Hz after 1 h, 3 h, and 6 h as mean ± range and marked by different letters. Different letters indicate statistically significant differences for each conching time determined via one factorial ANOVA (p < 0.05). Lower case letters indicate statistical differences between conching experiments at 1 h, capital letters indicate differences at 3 h, and bold lower case letters indicate differences at 6 h.

Generally, a decrease in the complex viscosity could be observed with increasing conching duration (Fig. 1). However, a remarkable difference was seen between the plastic masses conched in the presence of pre-charge and the masses without pre-charge. Without pre-charge, the complex viscosity continuously decreased comparing masses after 1 h, 3 h, and 6 h of conching at 60 °C forwards (Fig. 1A). During the conching experiments that were conducted in the presence of pre-charge, the complex viscosity was highest after 1 h in each conching experiment but the complex viscosity after 3 h and 6 h of conching was comparable (Fig. 1B, C, and D). Accordingly, the highest complex viscosity amongst all conching experiments was determined for conching without pre-charge at 60 °C forwards. The second highest complex viscosity was determined for the mass conched with pre-charge at 60 °C forwards (Fig. 1B).

Conching with pre-charge at 80 °C forwards and conching with pre-charge at 60 °C backwards reduced the complex viscosity most effectively (Fig. 1C and D). Both conditions resulted in the lowest complex viscosity determined after 3 h and 6 h of conching.

### 3.2 Odorant concentrations in the fat phase throughout conching

**Fig. 2:**
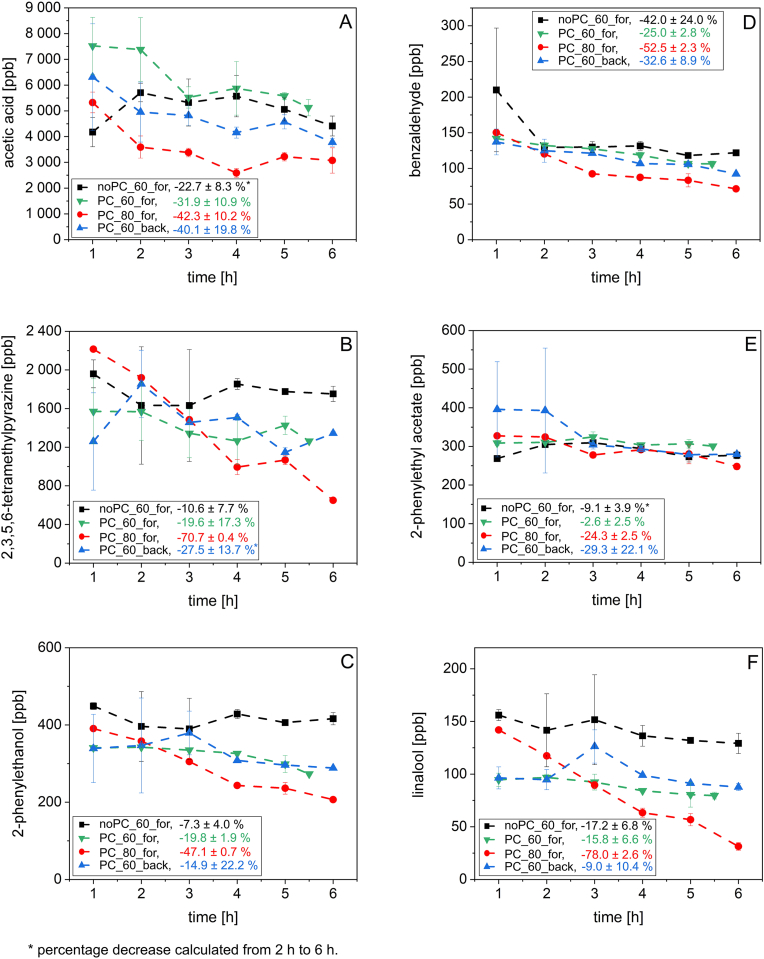
Concentrations of acetic acid (**A**), 2,3,5,6-tetramethylpyrazine (**B**), 2-phenylethanol (**C**), benzaldehyde (**D**), 2-phenylethyl acetate (**E**), and linalool (**F**) in the fat phase during conching (1–6 h) without pre-charge at 60 °C forwards (noPC_60_for), with pre-charge at 60 °C forwards (PC_60_for), with pre-charge at 80 °C forwards (PC_80_for), and with pre-charge at 60 °C backwards (PC_60_back). Additionally, the percentage decrease in concentration from 1 h to 6 h is provided. Error bars indicate the standard deviation from the mean.

Acetic acid, benzaldehyde, linalool, TMP, 2-phenylethanol, and 2-phenylethyl acetate were quantified in the fat phase in order to investigate the impact of different process parameters on the odorant concentration during conching (Fig. 2).

Acetic acid concentrations were found to be the highest among the selected odorants throughout the different conching trials. Across all conching experiments, acetic acid concentrations ranged from 2590 to 7520 ppb (Fig. 2A). After 1 h of conching, the highest concentration was observed during conching with pre-charge at 60 °C forwards (7520 ± 1110 ppb), and the lowest concentration was observed without pre-charge at 60 °C forwards (4180 ± 567 ppb). In general, acetic acid concentrations decreased throughout conching, except at the 1 h sampling point without pre-charge at 60 °C forwards. Remarkable was the effect of conching with pre-charge at 80 °C forwards on the acetic acid concentration. From 2 h onwards, the concentration remained consistently lower compared to conching at 60 °C. Throughout conching (1–6 h), the concentrations decreased by 42.3% with pre-charge at 80 °C forwards, by 40.1% with pre-charge at 60 °C backwards, and by 31.9% with pre-charge at 60 °C forwards. For conching without pre-charge at 60 °C forwards, a decrease by 22.7% (2–6 h) was recorded.

TMP was found in concentrations ranging from 649 to 2220 ppb (Fig. 2B). After the first hour of conching, TMP concentrations were found to be highest during conching with pre-charge at 80 °C forwards (2220 ± 15.8 ppb). Generally, the concentrations decreased continuously during conching, with the exception of conching at 60 °C backwards after 1 h. The concentration during conching backwards decreased by 27.5% (2–6 h). From 1 h to 6 h, the concentration decreased by 70.7% when conching with pre-charge at 80 °C forwards, by 19.6% during conching with pre-charge at 60 °C forwards, and by 10.6% during conching without pre-charge at 60 °C forwards.

2-Phenylethanol was quantified in amounts from 207 to 449 ppb (Fig. 2C). The concentration decrease (1–6 h) during conching was strongest with pre-charge at 80 °C forwards (by 47.1%). Conching with pre-charge at 60 °C forwards resulted in a concentration decrease by 19.8 %, and conching with pre-charge at 60 °C backwards reduced the concentration by 14.9%. The lowest concentration decrease was determined during conching without pre-charge at 60 °C forwards (by 7.3%).

Benzaldehyde was found in concentrations from 71.4 to 210 ppb (Fig. 2D). Similarly to acetic acid, TMP, and 2-phenylethanol, the concentration decreased strongest during conching (1–6 h) with pre-charge at 80 °C forwards (by 52.5%). Conching without pre-charge at 60 °C forwards resulted in a concentration decrease by 42.0%. During conching with pre-charge at 60 °C backwards, the benzaldehyde concentration was reduced by 32.6%, and with pre-charge at 60 °C forwards by 25.0%.

2-Phenylethyl acetate was found to be present in a concentration range from 248 to 396 ppb (Fig. 2E). Throughout conching (1–6 h), the concentration decrease was 29.3% during conching with pre-charge at 60 °C backwards and 24.3% while conching with pre-charge at 80 °C forwards. Conching with pre-charge at 60 °C forwards resulted in a decrease by 2.6%, while conching without pre-charge at 60 °C forwards caused the concentration to decrease by 9.1% (2–6 h).

Finally, linalool was the substance determined in the lowest concentrations amongst the investigated odorants. Linalool was found to be present in concentrations from 31.3 to 156 ppb (Fig. 2F). In comparison to the other odorants analyzed, the linalool concentrations after the first hour of conching varied significantly depending on the parameters applied. After 1 h, the linalool concentration was the highest after conching without pre-charge at 60 °C forwards (156 ± 5.23 ppb), followed by the concentration determined during conching with pre-charge at 80 °C forwards (142 ± 1.49 ppb). The lowest concentrations after 1 h of conching were comparable and were determined during conching with pre-charge at 60 °C backwards (96.4 ± 10.5 ppb), and conching with pre-charge at 60 °C forwards (94.6 ± 6.28 ppb). The decrease in linalool concentration during conching with pre-charge at 80 °C forwards was remarkable, representing the largest overall drop of 78.0% compared to the other odorants and conching parameters applied. During conching without pre-charge at 60 °C forwards, the linalool concentration decreased by 17.2% and conching with pre-charge at 60 °C forwards resulted in a decrease by 15.8%. Similar observations were made when conching with pre-charge at 60 °C backwards (−9.0%).

### 3.3 Distribution of selected odorants between fat and particle phase

**Fig. 3:**
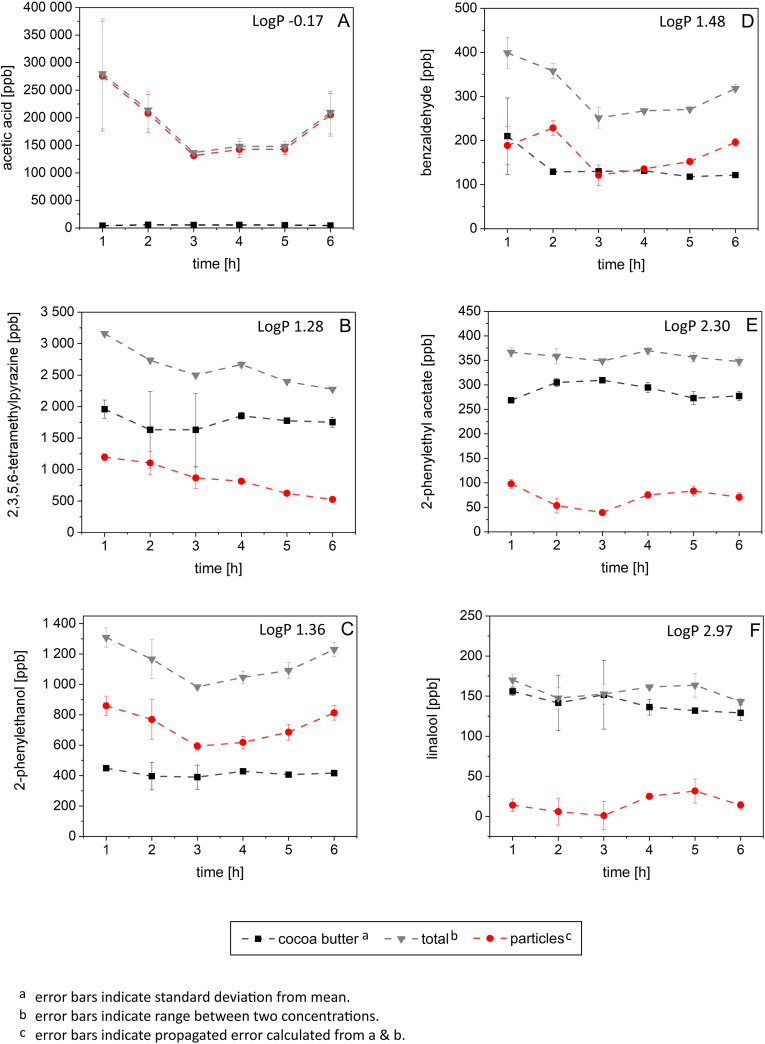
LogP and concentrations of acetic acid (**A**), 2,3,5,6-tetramethylpyrazine (**B**), 2-phenylethanol (**C**), benzaldehyde (**D**), 2-phenylethyl acetate (**E**), and linalool (**F**) in the fat phase, the particle phase, and the total plastic mass (1–6 h). Masses were conched without pre-charge at 60 °C forwards.

In order to investigate the odorant distribution between the fat and the particle phase throughout the conching process, the proportional concentrations of selected odorants in both phases were compared. The odorant distributions obtained from all conching experiments are given in the supplementary information. Acetic acid and 2-phenylethanol were found to be predominantly present in the particle phase independent of the different conching parameters applied. TMP, 2-phenylethyl acetate, and linalool were predominantly present in the fat phase during all conching experiments. For benzaldehyde, no clear tendency was observed as the odorant seemed to distribute almost equally between the fat and particle phases. In the following, the results of the odorant distribution will be presented and discussed exemplified for the concentrations determined in plastic masses conched at 60 °C forwards that did not include a pre-charge (Fig. 3).

The selected odorants were found to distribute between fat and particle phase according to their *n*-octanol/water partition coefficient (LogP), except for TMP (Table 2). In ascending order, acetic acid, 2-phenylethanol, benzaldehyde, TMP, 2-phenylethyl acetate, and linalool were increasingly located in the fat phase. Overall, the odorant distribution between fat and particle phases was comparable after 1 h and 6 h of conching, except for TMP, which showed a slight increase in fat phase enrichment after 6 h (+15.0%).

**Table 2:** Selected odorants, their *n*-octanol/water partition coefficient (LogP), and proportions of each odorant in the fat phase displayed as mean ± standard deviation after 1 h and 6 h of conching. For each odorant, different letters indicate a statistically significant difference (p < 0.05).

odorant	LogP^a^	proportion in fat phase [%]	
		1 h	6 h
acetic acid	−0.17	1.5 ± 0.6 ^a^	2.1 ± 0.4 ^a^
2,3,5,6-tetramethylpyrazine	1.28	62.0 ± 5.5 ^a^	77.0 ± 4.6 ^b^
2-phenylethanol	1.36	34.3 ± 1.8 ^a^	33.9 ± 1.9 ^a^
benzaldehyde	1.48	52.7 ± 25.2 ^a^	38.3 ± 1.6 ^a^
2-phenylethyl acetate	2.30	73.3 ± 2.3 ^a^	79.7 ± 4.0 ^a^
linalool	2.97	91.7 ± 5.9 ^a^	90.2 ± 9.6 ^a^

a (National Center for Biotechnology Information, 2024).

### 3.4 Significant influences of conching parameters on odorant concentrations & rheology

Overall statistically significant effects on the complex viscosity and each odorant concentration in the fat phase throughout the conching experiments were determined via four factorial ANOVA (supplementary information).

Four factorial ANOVA revealed that — with the exception of 2-phenylethyl acetate — all conching parameters had a statistically significant influence on the complex viscosity as well as on the odorant concentrations. For 2-phenylethyl acetate, a significant influence of conching time and direction of rotation was observed, resulting in lower concentrations with increasing conching time or after conching backwards. However, no clear influences of the various parameters on the 2-phenylethyl acetate concentration could be established. The results for the other odorants and the complex viscosity as influenced by the conching parameters are presented in the following.

In general, the **conching time** was statistically highly significant for the complex viscosity and the odorant concentrations (p < 0.001). The complex viscosity after 3 h and 6 h was found to be significantly different from the viscosity after 1 h of conching. However, there was no significant difference between the complex viscosity after 3 h and 6 h of conching. Analogously to the complex viscosity, the odorant concentrations at the early stage of conching generally differed significantly from those at the late stage of conching. The earliest effect of conching time was observed for benzaldehyde, where a significant effect on concentration was already evident after 2 h compared to 1 h conching time. Acetic acid concentration showed a significant difference from 3 h onwards compared to 1 h of conching, and TMP, 2-phenylethanol, and linalool from 4 h compared to 1 h conching time.

The impact of **pre-charge** in dependence of the conching time was statistically highly significant on the complex viscosity (p < 0.001). After 1 h of conching without pre-charge, the complex viscosity was significantly higher than after 1 h, 3 h, and 6 h of conching with pre-charge. Conching for 3 h without pre-charge was significantly higher than the complex viscosity after conching 3 h or 6 h with pre-charge. Throughout conching without pre-charge at 60 °C forwards, the highest concentrations of TMP, 2-phenylethanol, benzaldehyde, and linalool were detected. On the contrary, the highest acetic acid concentrations were determined during conching with pre-charge at 60 °C forwards (section 3.2). The presence of pre-charge during conching was determined to have a highly significant impact (p < 0.001) on the concentrations of acetic acid, TMP, 2-phenylethanol, and linalool. The benzaldehyde concentration was affected very significantly (p < 0.01). Conching with or without pre-charge was found to impact the acetic acid and benzaldehyde concentration significantly during the first hour of conching. Linalool concentrations after conching with and without pre-charge were significantly different from each other throughout the complete conching course (1–6 h).

The **conching temperature** had a very significant influence (p < 0.01) on the complex viscosity of the masses. The complex viscosity after conching at 60 °C for 1 h was determined to be significantly higher than the complex viscosity after conching at 80 °C for 3 h or 6 h. However, comparing the complex viscosity at the same conching time but at different conching temperature, no significant effects were observed. Throughout conching at elevated temperature, the lowest concentrations were detected for all odorants investigated. Consequently, statistical analysis revealed that conching at 80 °C had a highly significant influence (p < 0.001) on the acetic acid, 2-phenylethanol, benzaldehyde, and linalool concentrations. The TMP concentration was affected significantly (p < 0.05). Acetic acid and linalool were particularly affected by conching at 80 °C. Statistically significant concentration differences in dependence on the temperature were determined during almost the complete conching period but particularly at 2 h, 3 h, 4 h, and 5 h for acetic acid, and at 3 h, 4 h, 5 h, and 6 h for linalool. 2-phenylethanol concentrations at a conching temperature of 60 or 80 °C were likewise significantly different from 4 h on. For TMP, significantly different concentrations at 60 and 80 °C were observed in the beginning (1 h) and at the end (6 h) of conching.

The effect of the **conching direction** on the complex viscosity was determined to be highly significant (p < 0.001). After conching forwards for 1 h, the complex viscosity was significantly higher than the complex viscosity after conching backwards. The viscosity determined after conching backwards for 3 h or after 6 h was determined to result in a significantly different complex viscosity only from conching forwards for 1 h. The conching direction had a highly significant influence (p < 0.001) on the acetic acid and linalool concentrations, resulting in lower acetic acid concentrations after conching backwards. 2-Phenylethanol and benzaldehyde concentrations were affected very significantly (p < 0.01) by changing the conching direction. The TMP concentration was determined to be significantly (p < 0.05)different when conching backwards. Taking a closer look at the concentration differences in dependence of conching direction over time, significant differences in concentrations were particularly detected due to increasing conching time.

## 4 Discussion

### 4.1 Odorant concentrations quantified during conching

The selected odorants investigated in this study were generally quantified in lower concentrations in the fat phase compared to our previous study, which investigated the conching effect on a time-resolved basis using the same recipe on a smaller scale (Guckenbiehl et al., 2022). For instance, acetic acid was previously found at absolute concentrations approximately 2.4–2.9 times higher (25.5–63.2 ppm) than those determined in this study (8.93–25.9 ppm, supplementary information). The differences in concentration might stem from different batches of raw materials used for the experiments. Cocoa is a natural product and is therefore subjected to various elemental influences that can affect the aroma profile of cocoa (Gaspar et al., 2021). Further, different dimensions of conches were used, which suggested that odorant concentrations changed differently in the fat phase using a larger conche.

Acetic acid is known to have a vinegar-like aroma, which contributes to the overall aroma impression of chocolate but is undesired as an abundant compound (Afoakwa et al., 2009; Beckett et al., 2017). It is formed during cocoa bean fermentation by acetic acid bacteria, which oxidize ethanol derived from yeasts, and is reduced in concentration by roasting or conching (Jinap and Dimick, 1991; Schwan and Wheals, 2004; Ziegleder, 2017; Rottiers et al., 2019). In our study, acetic acid exhibited the overall highest concentrations in the fat phase of differently conched plastic masses, compared to the other selected odorants. Accordingly, acetic acid has previously been reported to have the highest concentration in cocoa or dark chocolate in comparison to other odorants (Chetschik et al., 2019; Frauendorfer and Schieberle, 2006, 2019).

TMP is reported to have roasted, green, mocha-like, or milky coffee-like impressions (Counet et al., 2002). It can be formed during roasting but also during cocoa bean fermentation and therefore be already present before heat treatment of the beans (Frauendorfer and Schieberle, 2008; Afoakwa et al., 2008b). Concerning the TMP levels detected in the fat phase in our study, Hinneh et al. (2019) found comparable amounts of TMP by quantifying a maximum concentration of 857 ppb in conventional and alternatively produced chocolates, whereas Liu et al. (2015) determined a comparable TMP concentration of 1140 ppb in dark chocolate.

2-Phenylethanol was described to elicit a rose-like aroma impression (Liu et al., 2015). The compound was already found to be present in unfermented cocoa beans but increases in concentration during cocoa bean fermentation, deriving from phenylalanine via the Ehrlich pathway of yeasts (Schwan and Wheals, 2004; Rottiers et al., 2019). Liu et al. (2015) and Seyfried and Granvogl (2019) determined 2-phenylethanol concentrations in dark chocolate, which were at least 7–8 times higher (1480–3380 ppb) than analyzed in the fat phase in our study. Possibly, 2-phenylethanol accumulated stronger in the cocoa particle phase (section 4.2), whereas the analysis of whole chocolate resulted in higher amounts of 2-phenylethanol.

The Strecker aldehyde benzaldehyde, which is obtained from phenylalanine (Granvogl et al., 2006; Escobar et al., 2021), has previously been characterized by an earthy, nutty (Owusu et al., 2012) or an almond-like aroma impression (Liu et al., 2015). Throughout our experiments, the benzaldehyde concentrations were consistent with the concentrations determined in dark chocolates by Hinneh et al. (2019) and Liu et al. (2015), who determined benzaldehyde concentrations of 82–377 ppb in dark chocolates.

2-Phenylethyl acetate was determined to exhibit a fruity (Schnermann and Schieberle, 1997) or a honey-like, flowery (Seyfried and Granvogl, 2019) impression. The aroma-active volatile was suggested to arise from yeast metabolism as well, possibly emerging by esterification of 2-phenylethanol (Aprotosoaie et al., 2016; Rottiers et al., 2019). In our study, 2-phenylethyl acetate was found to be present in amounts comparable to concentrations determined in two dark chocolates by Seyfried and Granvogl (2019). The authors detected up to 273 ppb in dark chocolate.

Linalool is described by a floral, fresh (Liu et al., 2015) or a flowery aroma (Counet et al., 2002). The substance is a biosynthesis product and its synthesis depends on factors like plant variety and growing conditions. It was therefore proposed to be an indicator for fine grade cocoa (Ziegleder, 1990). In this study, it was the substance determined in the lowest concentrations amongst the investigated odorants, which were about 2–4 times lower than amounts determined by Seyfried and Granvogl (2019).

### 4.2 Effects of conching on the odorant distribution within the chocolate mass

In the following, the results of the odorant distribution between fat and particle phase will be discussed exemplified for the concentrations determined in plastic masses conched without pre-charge at 60 °C forwards.

Generally speaking, a greater accumulation of the selected odorants in the fat phase was observed with increasing LogP, and thus with decreasing polarity, except for TMP. Despite the second lowest LogP of 1.28 among the six odorants studied, TMP was the third most abundant substance in the fat phase (Table 2). Previous findings revealed that TMP predominantly accumulated in the fat phase after reaching the equilibrium distribution between fat and cocoa particle phases (Guckenbiehl et al., 2024). Concerning the aroma changes during conching, Fischer et al. (2010) stated that concentrations of most aroma compounds decrease particularly in the fat phase during conching. However, in our study, TMP concentrations decreased more strongly in the particle phase (by 56.3%) than in the fat phase (by 10.6%), as revealed when comparing the TMP amount after 1 h and 6 h of plastic conching. During conching, TMP might already have been present in large amounts in the fat phase but was possibly further extracted from inside the cocoa particles by the fat and mechanical stress.

Since acetic acid was the most polar compound studied, the observed predominant presence of acetic acid in the polar particle phase appeared plausible. In terms of chocolate production, this means excess acetic acid needs to be removed from the surface and presumably also from the interior of the cocoa particles during conching. If acetic acid is primarily removed directly from the cocoa particles, the substance would probably evaporate particularly during the early stages of conching, as the particles are not yet completely coated by fat. This is in line with our results as a stronger decrease in total acetic acid concentration was observed at the beginning of the conching process and leveled off during the conching time.

Linalool represented the most non-polar compound, which had the highest LogP value among the selected odorants investigated throughout the conching process. Therefore, it seemed legitimate that linalool was predominantly present in the non-polar fat phase of the plastic masses. A nearly complete accumulation of linalool in the fat phase possibly resulted in the linalool concentrations being affected the most by varying conching parameters, particularly the conching temperature and the presence of pre-charge (section 3.4).

In our study, we observed an increase in both total and proportional concentration of acetic acid, 2-phenylethanol, and benzaldehyde in the particle phase after 6 h of conching without pre-charge at 60 °C forwards. This kind of concentration increase, previously undescribed, was unexpected. Based on our results, it remains uncertain whether the increase in concentration during conching is solely due to the formation of these odorants within the particle phase.

### 4.3 Different conching parameters affecting the odorant concentrations and the complex viscosity of plastic masses

The differently conched plastic masses were characterized rheologically and aroma-analytically to evaluate mechanisms occurring during conching in combination of both, aroma and texture changes.

Generally, a decrease in complex viscosity with increasing **conching time** could be attributed to the de-agglomeration effect that occurred during conching and released enclosed cocoa butter. By longer conching time, the solid particles in the plastic mass were increasingly coated with cocoa butter (Hoskin and Dimick, 1980), reducing solid-solid interactions and hence the viscosity. This result was in accordance with previous findings (Vivar-Vera et al., 2008; Urbańska et al., 2021; Guckenbiehl et al., 2022). Concerning the concentration of the selected odorants in the fat phase, a general decrease with increasing conching time was observed to varying degrees, mostly depending on the compound analyzed and the parameters applied. A dependence of the concentration decrease on the physico-chemical properties of selected compounds was shown and discussed in detail in a previous study (Guckenbiehl et al., 2022). In addition, relatively large deviations in compound concentrations were noticeable in the early stages of conching without pre-charge at 60 °C forwards, with pre-charge at 60 °C forwards, as well as at 60 °C backwards. An inhomogeneous distribution of the odorants could be the cause of this deviation and could have occurred due to an uneven distribution of the mechanical energy input within the conche, resulting in less mechanically stressed parts of the mass, particularly at the beginning of the conching process.

The **presence of pre-charge** was found to be an important factor throughout the conching experiments. The pre-charge consisted of residues from previously liquefied chocolate that contained more cocoa butter (39 g/100 g) than the plastic mass (29 g/100 g). Therefore, the fat content of plastic masses conched in the presence of pre-charge was slightly higher than it would have been without pre-charge. As the presence of pre-charge was accompanied by a gain in a well conched fat phase from the previous trials and possibly increased the fat content of the plastic mass, it is conceivable that the extra, well conched fat phase caused a dilution effect. Consequently, lower concentrations of TMP, 2-phenylethanol, benzaldehyde, and linalool were obtained in the fat phase when conching with pre-charge at 60 °C forwards, in comparison to conching without pre-charge. For linalool, the possible dilution effect appeared to be the strongest, which might be explained by the fact that the compound was mostly located in the fat phase (see 3.3). There was no significant effect on acetic acid due to the presence of pre-charge after 6 h, whereas this compound was present in the highest concentrations when conching with pre-charge at 60 °C forwards, hence with a higher fat content. A stronger accumulation of acetic acid in the cocoa butter with increasing fat content could be the consequence when conching with pre-charge, and has previously been observed in model systems (Guckenbiehl et al., 2024).

Very small changes in fat content have been described as having a dramatic effect on the viscosity of chocolate masses containing less than 32 g/100 g fat (Afoakwa et al., 2007). Each pre-charge consisted of well conched, de-agglomerated material that further reduced the viscosity of the plastic mass by providing free fat contributing to the flow. The presence of pre-charge may thus have led to more efficient embedding of the particles in the fat phase, accelerating the viscosity decrease. However, conching without pre-charge resulted in a weaker decrease in complex viscosity, which was possibly accompanied by less coated particles, enabling the volatilization of acetic acid directly from the cocoa particle phase compared to conching with pre-charge at 60 °C forwards. The acetic acid concentration and the complex viscosity therefore correlated negatively after 1 h of plastic conching. Conching with pre-charge was advantageous for a quicker attainment of a low viscosity of the chocolate mass, which could shorten the conching time by 3 h and therefore save energy cost. On the other hand, the acetic acid evaporation seemed less effective during conching with pre-charge, although not to a significant degree. However, desired odorants like TMP, 2-phenylethanol, benzaldehyde, and linalool appeared to volatilize to a lesser extent from the fat phase when conching without pre-charge.

For the development of the aroma during conching, maintaining high energy input by shearing a plastic mass with high viscosity is important (Beckett et al., 2017). The material inside the conche is sheared and smeared between the shear elements and the conche wall, and can reach an equilibrium viscosity that does not further decrease (Jolly et al., 2003; Beckett et al., 2017). The smearing of the material is particularly meaningful in the beginning of conching. The pre-charge present in the conche before material loading might cause the freshly loaded flakes to stick to the coated walls, narrowing the gap between shear elements and the conche wall, until the pre-charge is homogenously mixed into the plastic mass. A decreased gap between wall and shear elements is known to increase the shear effect (Jolly et al., 2003; Beckett et al., 2017). The increased shear possibly accelerated liquefaction by quicker de-agglomeration. Adhesion to the conche wall could also have increased the surface area available for aroma release. Therefore, additionally to the changing fat content, the pre-charge affecting the energy input into the mass could provide an explanation as to why the presence of the pre-charge had a significant influence on viscosity and aroma release in our study.

Generally, the odorant concentrations after 6 h were the lowest after conching at 80 °C forwards, which made the **temperature** the strongest effect on decreasing odorant concentrations (Fig. 2). A stronger decrease in concentration at higher conching temperature was plausible as higher temperatures and good aeration applied for several hours can increase the volatility of odorants and might therefore reinforce their release from the fat phase. This was also reflected in the boiling points of the substances investigated. For instance, acetic acid has the lowest boiling point (118 °C) and conching at 80 °C resulted in significantly lower acetic acid concentrations from 2 h to 5 h than conching at 60 °C. The same observation was made for 2-phenylethanol, which has a relatively high boiling point (218 °C). A lower complex viscosity caused by conching at elevated temperature appeared also plausible. Cocoa butter, and other fats and oils decrease in viscosity with increasing temperature (Timms, 1985; Landfeld et al., 2000; Shamsudin et al., 2006). Consequently, particles were coated more efficiently during conching at 80 °C, which resulted in an overall lower complex viscosity of the plastic mass. These findings are in accordance with results by Urbańska et al. (2021). The authors determined a decreasing viscosity of milk chocolate with increasing conching temperature (50–60 °C). Aligning the rheological and aroma-analytical results revealed that linalool and TMP, which were assumed to be predominantly located in the fat phase (section 4.2), showed a relatively high concentration in the first 2 h of conching, which was followed by a relatively strong decrease in concentration. During conching at 80 °C, a higher complex viscosity consequently correlates with higher concentrations in odorants that accumulated strongest in the fat phase. However, the TMP and linalool concentrations were lowest after 4 h of conching at 80 °C, while the complex viscosity did no longer change significantly. Apparently, this kind of concentration development during conching was also observed for 2-phenylethanol and benzaldehyde, but with a lower starting concentration (1 h) and therefore a lesser decline in concentration.

The **conching direction** was also an important process parameter influencing complex viscosity and odorant concentrations. A lower complex viscosity due to conching backwards is attributable to the construction of the shear elements. Conching backwards, which means working on the mass particularly with a flatter side of the shear element, results in higher shear forces and a greater mechanical stress on the mass. Meanwhile, conching forwards supports mixing and the discharge of moisture from the mass (Beckett et al., 2017). Changing the conching direction was found to be effective for removing excess acetic acid from the fat phase, as well as conching at 80 °C. In contrast, for all the other compounds investigated, conching at 60 °C backwards was interpreted to produce comparable concentrations to conching at 60 °C forwards (Fig. 2). Higher shear forces apparently supported the emission of the most polar substance investigated (acetic acid) from the non-polar fat phase. On the other hand, (rather) non-polar compounds remained in the fat phase, regardless of the mechanical stress applied by varying conching direction. Accordingly, the compound polarity apparently prevailed the mechanical impact on the odorant concentrations during conching forwards or backwards.

## 5 Conclusion

A deeper understanding of how aroma and texture changes are influenced by different impact factors during conching is given by this study, in which selected odorant concentrations in fat and particle phase as well as the complex viscosity of the plastic mass were monitored throughout conching. As expected, odorant concentrations in the fat phase decreased strongest during conching at elevated temperature. The highest odorant concentrations remained in the plastic mass predominantly after conching without pre-charge. Analogously to the aroma changes, the complex viscosity decreased strongest after conching at higher temperature and remained the highest after conching without pre-charge. Particularly for acetic acid, an increased complex viscosity in the beginning of conching appeared to support the evaporation of the odorant from the fat phase. Additionally, the presence of pre-charge accelerated the decrease in complex viscosity, achieving a constant complex viscosity after half of the conching time in every conching experiment conducted with pre-charge.

An increasing accumulation of less polar odorants in the fat phase has previously been shown in model systems and was confirmed in our study during real conching. Further, it was suggested that acetic acid and 2-phenylethanol were particularly located in the particle phase during conching, whereas TMP, 2-phenylethylacetate, and linalool were located in the fat phase.

Our data illustrate the complexity of the conching process and the need of meeting compromises during processing, as elevated conching temperatures, for example, accelerate the decrease of the complex viscosity, but also of the concentrations of value-adding odorants. Conching in a clean conche might promote the discharge of acetic acid, but also extends the conching time in order to obtain optimal flow properties of the mass.

In order to meet the industrial standards, where the process parameters are adjusted during the conching, the influence of a varied parameter within the same trial could be of interest to deepen the understanding of conching. In addition, the rotation speed can represent a crucial factor for aroma and texture changes as well, and should therefore be targeted in future studies.

## CRediT authorship contribution statement

**Yvonne Guckenbiehl:** Formal analysis, Investigation, Methodology, Validation, Visualization, Writing – original draft, Writing – review & editing. **Aurora Magdalena Morales Romero:** Formal analysis, Investigation. **Helen Haug:** Investigation, Methodology. **Eva Ortner:** Methodology, Supervision, Writing – review & editing. **Isabell Rothkopf:** Funding acquisition, Project administration, Supervision, Writing – review & editing. **Ute Schweiggert-Weisz:** Conceptualization, Supervision, Writing – review & editing. **Andrea Buettner:** Supervision, Writing – review & editing. **Susanne Gola:** Conceptualization, Project administration, Supervision, Writing – review & editing.

## Funding

The Industrial Collective Research is a programm financed by the Federal Ministry of Economic Affairs and Climate action (BMWK) in Germany, and this programm funding is possible based on a resolution of the German Parliament. The funding source is therefore the Federal Ministry of Economic Affairs and Climate Action (BMWK).

## Declaration of competing interest

The authors declare that they have no known competing financial interests or personal relationships that could have appeared to influence the work reported in this paper.

## Data Availability

Data will be made available on request.

## Appendix A Supplementary data

Odorant concentrations Multimedia component 1

Statistical Analysis - Rheology data Multimedia component 2

Statistical Analysis - Odorant concentrations fat phase Multimedia component 3

Table A.1 Multimedia component 4

Equation A.1 Equation A.1

## References

[bib1] Afoakwa E.O., Paterson A., Fowler M. (2007). Factors influencing rheological and textural qualities in chocolate – a review. Trends Food Sci. Technol..

[bib2] Afoakwa E.O., Paterson A., Fowler M. (2008). Effects of particle size distribution and composition on rheological properties of dark chocolate. Eur. Food Res. Technol..

[bib3] Afoakwa E.O., Paterson A., Fowler M., Ryan A. (2008). Flavor formation and character in cocoa and chocolate: a critical review. Crit. Rev. Food Sci. Nutr..

[bib4] Afoakwa E.O., Paterson A., Fowler M., Ryan A. (2009). Matrix effects on flavour volatiles release in dark chocolates varying in particle size distribution and fat content using GC-mass spectrometry and GC-olfactometry. Food Chem..

[bib5] Albak F., Tekin A.R. (2016). Variation of total aroma and polyphenol content of dark chocolate during three phase of conching. J. Food Sci. Technol..

[bib6] Aprotosoaie A.C., Luca S.V., Miron A. (2016). Flavor chemistry of cocoa and cocoa products – an overview. Compr. Rev. Food Sci. Food Saf..

[bib7] Augusto P.P.C., Bolini H.M.A. (2022). The role of conching in chocolate flavor development: a review. Compr. Rev. Food Sci. Food Saf..

[bib8] Beckett S.T. (2003). Is the taste of British milk chocolate different?. Int. J. Dairy Technol..

[bib9] Beckett S.T., Paggios K., Roberts I., Beckett S.T., Fowler M.S., Ziegler G.R. (2017). Beckett's Industrial Chocolate Manufacture and Use.

[bib10] Biehl B., Ziegleder G., Caballero B. (2003). Encyclopedia of Food Sciences and Nutrition.

[bib11] Bolenz S., Thiessenhusen T., Schäpe R. (2003). Fast conching for milk chocolate. Eur. Food Res. Technol..

[bib12] Chetschik I., Pedan V., Chatelain K., Kneubühl M., Hühn T. (2019). Characterization of the flavor properties of dark chocolates produced by a novel technological approach and comparison with traditionally produced dark Chocolates. J. Agric. Food Chem..

[bib13] Counet C., Callemien D., Ouwerx C., Collin S. (2002). Use of gas chromatography-olfactometry to identify key odorant compounds in dark chocolate. Comparison of samples before and after conching. J. Agric. Food Chem..

[bib14] Danzl W., Ziegleder G. (2014). Chocolate conching: aroma improvement by a changed flavour distribution. New Food.

[bib15] Engel W., Bahr W., Schieberle P. (1999). Solvent assisted flavour evaporation – a new and versatile technique for the careful and direct isolation of aroma compounds from complex food matrices. Eur. Food Res. Technol..

[bib16] Engeseth N.J., Ac Pangan M.F. (2018). Current context on chocolate flavor development – a review. Curr. Opin. Food Sci..

[bib17] Escobar S., Santander M., Zuluaga M., Chacón I., Rodríguez J., Vaillant F. (2021). Fine cocoa beans production: tracking aroma precursors through a comprehensive analysis of flavor attributes formation. Food Chem..

[bib18] Fischer A., Abubaker T., Hässelbarth A., Ullrich F., Blank I., Wüst M., Yeretzian C. (2010). Expression of Multidisciplinary Flavour Science: Proceedings of the 12^th^ Weurman Symposium; Interlaken, Switzerland, 2008.

[bib19] Frauendorfer F., Schieberle P. (2006). Identification of the key aroma compounds in cocoa powder based on molecular sensory correlations. J. Agric. Food Chem..

[bib20] Frauendorfer F., Schieberle P. (2008). Changes in key aroma compounds of Criollo cocoa beans during roasting. J. Agric. Food Chem..

[bib21] Frauendorfer F., Schieberle P. (2019). Key aroma compounds in fermented Forastero cocoa beans and changes induced by roasting. Eur. Food Res. Technol..

[bib22] Gaspar D.P., Chagas Junior G.C.A., Aguiar Andrade E. H. de, Nascimento L.D.d., Chisté R.C., Ferreira N.R., Da Martins L.H.S., Lopes A.S. (2021). How climatic seasons of the amazon biome affect the aromatic and bioactive profiles of fermented and dried cocoa beans?. Molecules.

[bib23] Granvogl M., Bugan S., Schieberle P. (2006). Formation of amines and aldehydes from parent amino acids during thermal processing of cocoa and model systems: new insights into pathways of the strecker reaction. J. Agric. Food Chem..

[bib24] Guckenbiehl Y., Martin A., Ortner E., Rothkopf I., Schweiggert-Weisz U., Buettner A., Naumann-Gola S. (2022). Aroma-active volatiles and rheological characteristics of the plastic mass during conching of dark chocolate. Food Res. Int..

[bib25] Guckenbiehl Y., Ortner E., Rothkopf I., Schweiggert-Weisz U., Ziegleder G., Buettner A., Naumann-Gola S. (2024). Distribution and transition of aroma-active compounds in dark chocolate model systems under conching conditions. Food Chem..

[bib26] Hinneh M., van de Walle D., Tzompa-Sosa D.A., Haeck J., Abotsi E.E., Winne A. de, Messens K., van Durme J., Afoakwa E.O., Cooman L. de, Dewettinck K. (2019). Comparing flavor profiles of dark chocolates refined with melanger and conched with Stephan mixer in various alternative chocolate production techniques. Eur. Food Res. Technol..

[bib27] Hoskin J.M., Dimick P.S. (1980). Observation of chocolate during conching by scanning electron microscopy and viscometry. J. Food Sci..

[bib28] Jinap S., Dimick P.S. (1991). Effect of roasting on acidic characteristics of cocoa beans. J. Sci. Food Agric..

[bib29] Jolly M.S., Blackburn S., Beckett S.T. (2003). Energy reduction during chocolate conching using a reciprocating multihole extruder. J. Food Eng..

[bib30] Landfeld A., Novotna P., Strohalm J., Houska M., Kyhos K. (2000). Viscosity of cocoa butter. Int. J. Food Prop..

[bib31] Liu J., Liu M., He C., Song H., Guo J., Wang Y., Yang H., Su X. (2015). A comparative study of aroma-active compounds between dark and milk chocolate: relationship to sensory perception. J. Sci. Food Agric..

[bib32] National Center for Biotechnology Information (2024). PubChem.

[bib33] Owusu M., Petersen M.A., Heimdal H. (2012). Effect of fermentation method, roasting and conching conditions on the aroma volatiles of dark chocolate. J. Food Process. Preserv..

[bib34] Owusu M., Petersen M.A., Heimdal H. (2013). Relationship of sensory and instrumental aroma measurements of dark chocolate as influenced by fermentation method, roasting and conching conditions. J. Food Sci. Technol..

[bib35] Rottiers H., Tzompa Sosa D.A., Winne A. de, Ruales J., Clippeleer J. de, Leersnyder I. de, Wever J. de, Everaert H., Messens K., Dewettinck K. (2019). Dynamics of volatile compounds and flavor precursors during spontaneous fermentation of fine flavor Trinitario cocoa beans. Eur. Food Res. Technol..

[bib36] Schnermann P., Schieberle P. (1997). Evaluation of key odorants in milk chocolate and cocoa mass by aroma extract dilution analyses. J. Agric. Food Chem..

[bib37] Schwan R.F., Wheals A.E. (2004). The microbiology of cocoa fermentation and its role in chocolate quality. Crit. Rev. Food Sci. Nutr..

[bib38] Seyfried C., Granvogl M. (2019). Characterization of the key aroma compounds in two commercial dark chocolates with high cocoa contents by means of the sensomics approach. J. Agric. Food Chem..

[bib39] Shamsudin R., Mohamed I.O., Nooi T.S. (2006). Rheological properties of cocoa butter substitute (CBS): effects of temperature and characteristics of fatty acids on viscosity. J. Food Lipids.

[bib40] Timms R.E. (1985). Physical properties of oils and mixtures of oils. J. Am. Oil Chem. Soc..

[bib41] Toker O.S., Palabiyik I., Pirouzian H.R., Aktar T., Konar N. (2020). Chocolate aroma: factors, importance and analysis. Trends Food Sci. Technol..

[bib42] Urbańska B., Kowalska H., Szulc K., Ziarno M., Pochitskaya I., Kowalska J. (2021). Comparison of the effects of conching parameters on the contents of three dominant flavan3-ols, rheological properties and sensory quality in chocolate milk mass based on liquor from unroasted cocoa beans. Molecules.

[bib43] Vivar-Vera G., Torrestiana-Sanchez B., Monroy-Rivera J.A., La Brito-De Fuente E. (2008). Chonching: rheological and structural changes of chocolate mass. Dtsch. Lebensm.-Rundsch..

[bib44] Ziegleder G. (1990). Linalool contents as characteristic of some flavor grade cocoas. Z. Lebensm. Unters. Forsch..

[bib45] Ziegleder G., Beckett S.T., Fowler M.S., Ziegler G.R. (2017). Beckett's Industrial Chocolate Manufacture and Use.

[bib46] Ziegleder G., Balimann G., Mikle H., Zaki H. (2003). New insights into conching: Part 1–3. Süsswaren.

