# Supplementary material for: Conching of dark chocolate – Processing impacts on aroma-active volatiles and viscosity of plastic masses
Source: Curr Res Food Sci. 2024 Oct 31;9:100909. doi: 10.1016/j.crfs.2024.100909 (PMC11585642; doi:10.1016/j.crfs.2024.100909)
Supplement: Table A.1 [file mmc4.pdf]

Table A.1

Ions (*m/z*) of selected analytes and the respective isotope-labeled standards used for quantification.

Unlabeled analyte	Respective isotope-labeled standard	Mass trace analyte [*m/z*]	Mass trace isotope-labeled standard [*m/z*]
acetic acid	$[^{13}C_2]$ -acetic acid	60	62
benzaldehyde	$[^2H_5]$-benzaldehyde	106	111
(R,S)-(±)-linalool	$[^2H_{4\text{-}5}]$-(R,S)-(±)-linalool	93	97/98
2,3,5,6-tetra-methylpyrazine	$[^2H_{12}]$-2,3,5,6-tetramethylpyrazine	136	148
2-phenylethanol	$[^2H_5]$-2-phenylethanol	122	127
2-phenylethyl acetate	$[^2H_5]$-2-phenylethyl acetate	104	109
