# Supplementary material for: Conching of dark chocolate – Processing impacts on aroma-active volatiles and viscosity of plastic masses
Source: Curr Res Food Sci. 2024 Oct 31;9:100909. doi: 10.1016/j.crfs.2024.100909 (PMC11585642; doi:10.1016/j.crfs.2024.100909)
Supplement: Equation A.1 [file mmc5.pdf]

$$\Delta c_{part} = \sqrt{\Delta c_{total}^2 + \left(-p_{fat} \cdot \Delta c_{fat}\right)^2} \qquad \text{(Eq. A.1)}$$

With Δc_{part}: propagated error from the mean proportional concentration of selected odorants in the particle phase [ppb]

Δc_{fat}: standard deviation from the mean proportional concentration of selected odorants in the fat phase [ppb]

Δc_{total}: range of total concentration of selected odorants [ppb]

p_{fat}: proportion of fat phase in the plastic mass [%]
